# Health outcomes and adherence to a healthy lifestyle after a multimodal intervention in people with multiple sclerosis: Three year follow-up

**DOI:** 10.1371/journal.pone.0197759

**Published:** 2018-05-23

**Authors:** Claudia H. Marck, Alysha M. De Livera, Chelsea R. Brown, Sandra L. Neate, Keryn L. Taylor, Tracey J. Weiland, Emily J. Hadgkiss, George A. Jelinek

**Affiliations:** 1 Neuroepidemiology Unit, the Melbourne School of Population and Global Health, the University of Melbourne, Victoria, Australia; 2 Disability and Health, the Melbourne School of Population and Global Health, the University of Melbourne, Victoria, Australia; 3 City of Whittlesea, Victoria, Australia; Heinrich-Heine-Universitat Dusseldorf, GERMANY

## Abstract

**Background:**

Modifiable risk factors such as smoking and sedentary lifestyle adversely affect multiple sclerosis (MS) progression. Few multimodal behavioural interventions have been conducted for people with MS, and follow-up beyond 1 year is rare for lifestyle interventions. This study assessed adoption and adherence to healthy lifestyle behaviours and health outcomes 3 years after a lifestyle modification intervention, using generalized estimating equation models to account for within-participant correlation over time.

**Methods:**

95 people with MS completed baseline surveys before participating in 5-day MS lifestyle risk-factor modification workshops. 76 and 78 participants completed the 1-year and 3-year follow-up surveys respectively. Mean age at 3-year follow-up was 47 years, 72% were female, most (62.8%) had MS for 5 years or less, and 73% had relapsing remitting MS (RRMS).

**Results:**

Compared to baseline, participants reported clinically meaningful increases in physical (mean difference (MD): 8.0, 95% Confidence Interval (CI): 5.2–10.8) and mental health (MD: 9.2, CI: 5.8–12.6) quality of life (QOL) at 1-year, and physical (MD: 8.7, CI: 5.3–12.2) and mental health (MD: 8.0, CI: 4.2–11.8) QOL at 3-year follow-up. There was a small decrease in disability from baseline to 1-year follow-up (MD: 0.9, CI: 0.9,1.0) and to 3-year follow-up (MD: 1.0, CI: 0.9,1.0), which was not clinically meaningful. Of those with RRMS, compared to baseline, fewer had a relapse during the year before 1-year follow-up (OR: 0.1, CI 0.0–0.2) and 3-year follow-up (OR: 0.15, CI 0.06–0.33). Participants’ healthy diet score, the proportion meditating ≥1 hours a week, supplementing with ≥ 5000IU vitamin D daily, and supplementing with omega-3 flaxseed oil increased at 1-year follow-up and was sustained, although slightly lower at 3-year follow-up. However, there was no evidence for a change in physical activity and not enough smokers to make meaningful comparisons. Medication use increased at 1-year follow-up and at 3-year follow-up.

**Conclusion:**

The results provide evidence that lifestyle risk factor modification is feasible and sustainable over time, in a small self-selected and motivated sample of people with MS. Furthermore, participation in a lifestyle intervention is not associated with a decrease in MS medication use.

## Introduction

The onset of multiple sclerosis (MS), a chronic inflammatory and neurodegenerative disorder, can be explained by an interplay of genetic and environmental risk factors, including modifiable lifestyle factors[1]. The progression of MS, usually characterised by relapses and/or accumulating disability, is highly variable. Pharmacological treatment for MS has improved, but there is risk for side effects, and there are very limited options for people with progressive MS[2, 3]. A recent study has started to uncover some genetic drivers of MS progression[4], but strong evidence already exists that shows that modifiable risk factors such as smoking and low vitamin D levels affect MS progression[5]. These and other modifiable risk factors, such as regular physical activity, stress reduction, healthy weight, and normal blood lipid profiles are associated with other important health outcomes including quality of life (QOL), which is increasingly used as an outcome in MS trials.

In recent years research interest in modifiable lifestyle risk factors for MS risk and progression has increased, and some have postulated that MS may be a lifestyle disease[6]. However, there is a gap in the translation and implementation of research findings and, in fact, people with MS (PwMS) have less healthy lifestyles than the general population [7]. Modification of these risk factors provides a promising preventive medicine strategy to manage MS progression with the potential to decrease the burden on PwMS, their families and carers, as well as the health system [8].

Many PwMS are motivated to modify their risk factors for disease progression. A survey showed that 85% of 199 surveyed PwMS in the United States were willing to try a dietary intervention for at least 3 months[9]. However, few trials measure adherence, for example lifestyle changes, and outcomes beyond 12 months[10]. Furthermore, while many interventions in MS have focussed on a single health behaviour such as diet or physical activity, there is some evidence that a multimodal intervention, targeting a variety of risk-factors at once, may have benefits over and above the sum of benefits[11]. Very few multimodal interventions have been reported in MS, and these were on fewer than 20 PwMS [12, 13].

The aim of this study was to assess adherence to healthy lifestyle recommendations and health outcomes 3 years after a multimodal lifestyle risk-factor intervention in PwMS.

## Methods

This study is part of a larger study evaluating the effects of a lifestyle modification intervention. A subgroup was invited to complete this 3-year follow up. We have previously reported QOL results from a different subgroup using the same intervention[14, 15]. Below we describe the methodology using the template for intervention description and replication (TIDieR) guide [16].

### Participants and recruitment

The intervention was advertised on MS websites with a focus on lifestyle, and participants enrolled without needing referral from a clinician. A few weeks before the workshop, all participants were asked to enrol in this study with longitudinal follow up if they were 18 years or older, by signing a consent form and completing an online or hardcopy baseline survey. Participants who had previously participated in the workshops were excluded from the study.

### The intervention

The intervention—5-day residential group workshops—promoted a very low saturated fat, plant-based whole food plus seafood diet, omega-3 supplementation, regular exercise, non-smoking, adequate vitamin D levels and stress reduction techniques such as meditation (Table 1). These workshops commenced in 2002 and are ongoing. The six workshops from which participants for this study were recruited were run between March 2012 and May 2013 and held in a purpose-built retreat facility that was designed and used from the early 1980s for cancer retreats; MS retreats were added in 2002. The venue consisted of individual and shared accommodation, meeting rooms, consulting rooms, and a hospitality-grade kitchen, with permanent therapeutic, cooking and administration staff. It was wheelchair accessible and located in rural Victoria, Australia. There were five intervention facilitators for the MS retreats, specifically the Therapeutic Director (with post-graduate qualifications and experience in counselling), the retreat leader (a medical professor with a strong academic and personal interest in MS), a psychiatrist (at the time in training to become retreat leader), and two facilitators (both trained and experienced in meditation instruction and counselling). Two of the five facilitators were diagnosed with MS, and all facilitators had extensive knowledge about MS.

**Table 1:** Lifestyle recommendations of the intervention.

Lifestyle factor	Recommendation
Diet	A plant-based wholefood diet plus seafood, with very low saturated fat (<20g/day) No dairy, meat, palm or coconut oil
Dietary supplementation	Omega-3 fatty acid supplements: 20-40mls of fish or flaxseed oil daily; can be omitted on days when oily fish is eaten Optional B group vitamins or B12 supplement if needed
Physical activity	20–30 minutes around five times a week, preferably outdoors
Smoking	No tobacco smoking and avoid passive smoking
Vitamin D	Sunlight 15 minutes daily 3–5 times a week as close to all over body exposure as practical Vitamin D3 supplement of at least 5000IU daily, adjusted to blood level Aim to keep blood level of vitamin D high, between 150-225nmol/L (may require up to 10,000IU daily)
Meditation	30 minutes daily

The facilitators delivered evidence-based information regarding lifestyle-related risk factor modifications associated with improved health outcomes in MS and practical advice on incorporating these into daily life in a face-to-face group setting. S2 Fig outlines the intervention program. The focus was on improving long-term physical and mental health. Sessions included presentations regarding the available evidence of how the lifestyle factors are associated with health outcomes in MS and the underlying mechanism of action, group discussions to clarify any questions and to share ways to overcome barriers to a healthy lifestyle, and practical sessions such as group meditations and exercises (tailored to level of ability). Further information sessions were held that included evidence-based information regarding MS medication, support services, how to disclose information regarding MS to family, friends and colleagues, and how to minimize risk of MS to family members, including risks of active and passive smoking, lack of sun exposure and low vitamin D, and overweight and obesity. In total there were 32 hours of formal contact between facilitators and participants, and considerable informal contact over meal and tea breaks, and out of session. Virtually all participants attended all sessions of every retreat, with only occasional absence due to medical conditions, which was communicated in advance to session facilitators. All interactions with retreat participants were focussed on positivism, wellbeing and empowerment. Groups of up to 35 participants consisted of mostly PwMS with some support people (partners or family members). Participants were provided with a take-home folder including printed information and presentation slides and the book “Overcoming MS” from the lead facilitator (GJ) [17]. Online support groups were created for those wishing to participate, but no data on engagement in this are available. No adverse events were reported.

Participant fees varied depending on level of accommodation, and also covered food and other expenses, coordinated through a not-for-profit charity Overcoming Multiple Sclerosis. More information regarding the content of the recommendations including the evidence base can be found on their website[18].

### Data collection and follow-up

Participants were invited by email for follow-up survey completion at one and three years post-intervention, and reminded by phone or follow-up emails. Data were mostly collected using online survey methodology, and some paper surveys sent through mail and entered in manually by the researchers. The survey took approximately 30–40 minutes to complete and participants were able to take breaks and return to the online survey at a later time if needed.

### Materials

Psychometrically sound and previously tested questionnaires were included in the survey, supplemented by researcher-devised items where existing tools were not available. These are summarised and listed below. All measures were self-reported. S1 Fig includes the survey questions used in this study.

#### Health outcomes

Participants self-reported the number of doctor-diagnosed relapses experienced in the previous year. For purpose of analysis, participants were categorised as having had a relapse in the previous year, or not. Disability was assessed using the validated and widely used tool Multiple Sclerosis Impact Scale Physical Component (MSIS-20), (with 8 point change seen as clinically meaningful[19]). Health-related QOL was measured with the Multiple Sclerosis Quality of Life survey (MSQOL-54) [20], an extensively validated measure. We report the mental and physical health composite scores, with a 5 point change in one of the composite scores seen as clinically meaningful[21].

#### Measures of modifiable lifestyle factors

Diet was assessed using a slightly modified version of The Diet Habits Questionnaire (DHQ, assessing quality of diet on a 0–100 scale with 100 being the healthiest diet possible)[22]. Exercise was assessed using the International Physical Activity Questionnaire Short Form (IPAQ) [23], a validated 7-day recall of frequency and duration of physical activity; which categorises people’s level of physical activity into low, moderate and high. For the purpose of analysis this was collapsed into low vs. moderate/high. Participants were asked to indicate current smoking status (yes, no); their average daily dose of vitamin D supplementation; whether they took omega-3 supplements, and which type, and weekly frequency and duration of meditation. A minimum of 5000IU per day of Vitamin D supplementation was recommended, and this was used as a cut off for the purpose of data analysis. Flaxseed oil supplementation was the recommended omega-3 supplementation, and this was used in analysis (vs no flaxseed oil supplementation) regardless of dose, as this is difficult to measure. Time spent meditating each week was categorised into <1 hour vs ≥ hour per week.

Other recorded variables were age, gender, partner status (partnered vs not), employment status (full time, part time, not employed), level of education (university level or below), and current medication use (a list of disease modifying drugs, with participants asked to indicate current use).

### Consent procedure

Written consent was obtained from all participants included in the study at baseline, and if completing paper follow-up surveys. At follow-up a participant information form was attached to the email invitation and a statement on the first page of the online survey reiterated that completion of the online survey was taken as consent to participate. The study was approved by the Health Sciences Human Ethics sub committee of University of Melbourne (1545287).

### Statistical analysis

Data were analysed using Stata, V14 (StataCorp, College Station, Texas, USA) and R 3.13 (R Foundation for Statistical Computing, Vienna, Austria). We report descriptive statistics of demographics, lifestyle behaviours and health outcomes in Table 2. Continuous data are summarised by means and standard deviations, and categorical data are summarised by percentages with frequencies. Skewed data were log transformed prior to analyses. To explore the change in each variable at each follow-up compared to baseline, a generalized estimating equation model was fitted to each variable, with an unstructured working correlation matrix to account for within individual correlations [24]. In Table 3, we present the results obtained from the complete case analysis, that is the analysis restricted to respondents with available data. As a sensitivity analysis, we used multiple imputation with auxiliary variables to account for missing values in our main outcome variables. For multiple imputation, Fully Conditional Specification [25] with a single imputation model consisting of the variables in the analysis model and auxiliary variables shown in S1 Table was used [26]. The results from fifty created multiply imputed datasets were combined using Rubin’s rules[26] and we present these results in S2 Table.

**Table 2:** Sample description over time.

Health outcomes	Baseline	1 year follow-up	3 year follow-up
Level of disability (MSIS)*^1^	32.7 (1.4)	29.7 (1.4)	30.9 (1.5)
Mental health QOL*^2^	66.8 (20.0)	77. 9 (15.6)	74.9 (19.0)
Physical health QOL*^3^	62.5 (19.9)	72.5 (19.5)	73.4 (19.3)
Any relapses in previous calendar year (those with relapsing-remitting MS only)			
Yes	47/67 (70.2)	9/57 (15.8)	14/55 (25.5)
**Health behaviours**			
Diet score*^4^	86.1 (9.7)	90.7 (6.2)	89.0 (6.0)
Meditation^#^			
< 1 hour a week	32/90 (35.6)	8/74 (10.8)	16/74 (21.6)
≥ 1 hour a week	58/90 (64.4)	66/74 (89.2)	58/74 (78.4)
Vitamin D ≥ 5000 IU daily ^#^			
	42/89 (47.2)	52/71 (73.2)	50/72 (69.4)
Flaxseed oil supplementation ^#^			
Yes	33/89 (37.08)	61/74 (82.43)	56/74 (75.68)
Moderate or high exercise^#^			
Yes	62/95 (65.3)	54/74 (73.0)	51/65 (78.5)
Disease modifying drug use^#^			
Yes	51/89 (57.3)	53/74 (71.6)	52/75 (69.3)
**Demographic variables**			
Gender^#^			
Male	26/95 (27.4)	22/76 (29.0)	22/78 (28.2)
Female	69/95 (72.6)	54/76 (71.1)	56/78 (71.8)
Age (years)*	44.0 (10.5)	44. 5 (10.6)	47.4 (10.8)
Partnered^#^			
Yes	73/95 (76.8)	59/74 (79.7)	57/76 (75.0)
University level education^#^			
Yes	68/95 (71.6)	59/75 (78.7)	57/76 (75.0)
Employment^#^			
Full time	35/95 (36.8)	30/73 (41.1)	23/76 (30.3)
Part time	35/95 (36.8)	26/73 (35.6)	30/76 (39.5)
Not employed	25/95 (26.3)	17/73 (23.3)	25/76 (32.9)
Disease duration			
Less than 2 years	47/95 (49.5)	19/76 (25.0)	0/78
2–5 years	18/95 (19.0)	38/76 (50.0)	49/78 (62.8)
6–10 years	17/95 (17.9)	10/76 (13.2)	9/78 (11.5)
11 or more years	13/95 (13.7)	9/76 (11.8)	20/78 (25.6)
Type of MS			
Relapsing remitting	68/95 (71.6)	58/75 (77.3)	56/77 (72.7)
Progressive	9/95 (9.5)	5/75 (6.7)	5/77 (6.5)
Benign/other	18/95 (19.0)	12/75 (16.0)	16/77 (20.8)

* mean (sd)

^#^Number (%)

^1^Sample size at baseline (N_1_) = 89 1-year follow-up (N_2_) = 72, 3-year follow-up (N_3_) = 72

^2^(N_1_ = 93, N_2_ = 73, N_3_ = 74)

^3^(N_1_ = 94, N_2_ = 74, N_3_ = 67)

^4^(N_1_ = 83, N_2_ = 65, N_3_ = 73)

**Table 3:** Changes in health outcomes and health behaviours over time using generalized estimating equation models to account for within-participant correlation over time.

Health outcomes		Mean difference* or Odds ratio	95% CI	p-value
Level of disability (MSIS)*	Baseline	Reference		
	I year	0.9	(0.9,1.0)	0.02
	3 year	1.0	(0.9,1.0)	0.08
Mental health QOL*	Baseline	Reference		
	I year	9.2	(5.8,12.6)	<0.001
	3 year	8.0	(4.2,11.8)	<0.001
Physical health QOL*	Baseline	Reference		
	I year	8.0	(5.2,10.8)	<0.001
	3 year	8.7	(5.3, 12.2)	<0.001
Any relapses (participants with RRMS only)	Baseline	Reference		
	I year	0.1	(0.0, 0.2)	<0.001
	3 year	0.2	(0.1, 0.3)	<0.001
**Health behaviours**				
Diet score*	Baseline	Reference		
	I year	4.5	(2.9, 6.1)	<0.001
	3 year	2.9	(1.0, 4.7)	<0.001
Meditation ≥ 1 hour a week	Baseline	Reference		
	I year	4.5	(2.2, 9.2)	<0.001
	3 year	2.0	(1.1, 3.7)	0.03
Vitamin D supplementation ≥ 5000 IU daily	Baseline	Reference		
	I year	3.4	(1.9, 6.0)	<0.001
	3 year	2.4	(1.4, 4.2)	<0.001
Flaxseed oil supplementation	Baseline	Reference		
	I year	7.5	(3.8, 14.7)	<0.001
	3 year	5.4	(3.1, 9.4)	<0.001
Moderate or high exercise	Baseline	Reference		
	I year	1.4	(0.8, 2.5)	0.2
	3 year	2.0	(1.0, 4.0)	0.07
Disease modifying drug use	Baseline	Reference		
	I year	1.8	(1.1, 2.8)	0.01
	3 year	1.8	(1.0, 3.0)	0.04

* Mean differences and 95% confidence intervals are presented.

## Results

Of 134 participants who participated for the first time in one of six workshops 39 (29.1%) either did not have a confirmed MS diagnosis at baseline or follow-up (e.g., clinically isolated syndrome), or did not complete a baseline survey, and were therefore excluded from the study. 95 (70.9%) consented and completed the baseline survey, out of those, 76 (80.0%) and 78 (82.1%) completed 1 and 3 year follow-up, respectively, but completion varied per survey item (Table 2).

**Fig 1:**
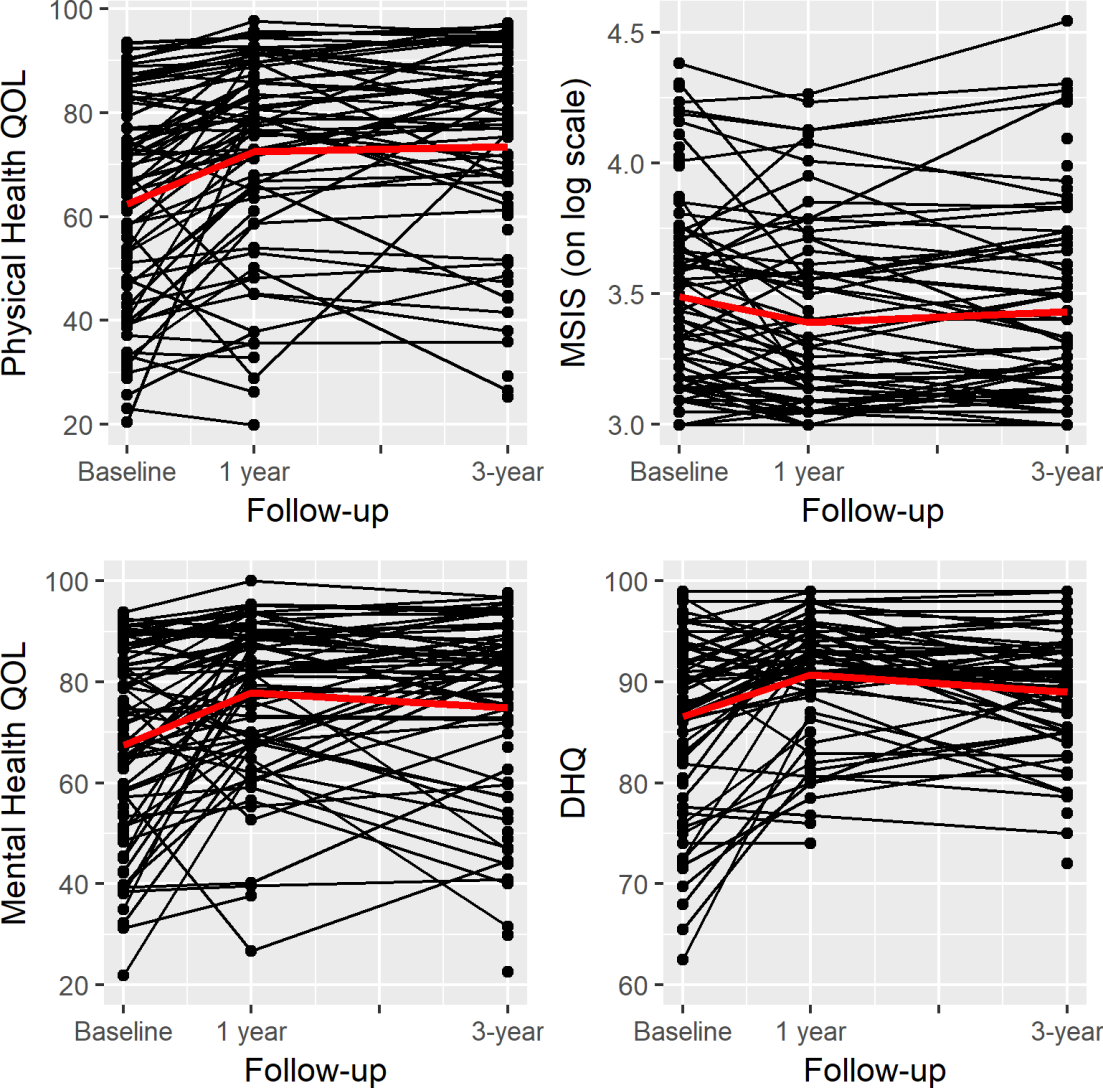
Changes observed over time in physical and mental health quality of life, disability, and diet. Quality of life was measured with the MSQOL-54, disability was measured by MSIS on log scale, and diet was measured by the DHQ.

Improvements in health outcomes were seen 1 year post-intervention, and were mostly sustained 3 years post intervention, with the exception of MSIS at 3 years. All health behaviours improved 1 year post-intervention, and were mostly sustained at 3 years post-intervention, except physical activity, which improved but non-significantly from baseline to 1 and 3 years post-intervention (Fig 1 and Table 3).

## Discussion

Patient self-management is recognised as a key to effective and efficient management of chronic disease. In chronic disease, health care professionals ideally assist patients to develop skilled health practices and the patient takes a more active role acquiring and applying relevant knowledge to their own circumstances[27]. Our multimodal lifestyle risk factor intervention in PwMS, delivered by health care professionals who aimed to assist participants in acquiring and applying relevant knowledge, was developed to assist in patient self-management. Results of our 3 year follow up showed improvements in health behaviours and outcomes, in line with results previously published on other samples only assessing QOL changes[14, 15].

From the 1960s onwards, with the rising incidence of chronic disease, a patient’s ability to function (not just the status of their disease) was recognised as an important outcome, and a patient’s attitude towards and perceptions of his or her situation were recognised as determinants of function. Continued engagement with and persistence in efforts to overcome problems related to the disease and positive self-perceptions resulted in both better function and survival. These observations led to the development of the concept of quality of life and the recognition of its importance in outcomes of chronic diseases[27]. Self-reported QOL has recently been recognized as one of the most important health outcomes for PwMS[28]. Importantly, in our study, participants reported clinically relevant increases (>5 points[21]) in both physical and mental health QOL, which were sustained at 3 years post-intervention. Other group interventions in PwMS have shown improvements in QOL by improving optimism in PwMS [29], and although we did not measure this in our sample, the positive outlook of the facilitators (some diagnosed with MS themselves), and focus on empowerment may have been important components of the intervention.

Of those participants with RRMS, the proportion reporting a relapse in the year post-intervention decreased dramatically compared to the year pre-intervention (from 70% to 16%). The proportion experiencing a relapse in the year prior to 3-year follow-up increased slightly to 26% but was still markedly and significantly lower than at baseline. It is possible that participants who had just experienced a relapse enrolled into the intervention for that reason, and this may have inflated the number of relapses reported at baseline, a phenomenon also recognised in another study[30]. This study on over 17,000 PwMS from the MSBase registry reported that the annual relapse rate gradually decreased from 0.5 at 2 years post-diagnosis to 0.1 at 40 years post-diagnosis[30].

Participants reported a small, not clinically meaningful, decrease in disability (below 7.5 or 8 points change in MSIS [19, 31]). Disability accumulation can occur at different rates in PwMS, but usually slowly over time. However, stable disability is clearly preferred over deterioration. An association between self-reported overall health-promoting behaviour, including physical activity, nutrition and spiritual growth, and slower disability progression has previously been observed in a cross-sectional study[32].

The intervention successfully changed behaviours but, more importantly, maintained these healthier behaviours over time although a slight decrease in health behaviours was seen at 3-year follow-up compared to 1 year follow-up. The one exception was physical activity. This may reflect the small proportion of time spent during the intervention on physical activity, and this has since been improved after feedback from participants. The efficacy of physical activity interventions may also differ by MS type, disability status, and weight status [33].

While the general population perceives many barriers to maintaining a healthy lifestyle [34], common MS symptoms and comorbidities such as depression, increasing disability and pain may further hamper these efforts [35]. However the successful and maintained changes in health behaviours illustrate how, despite obvious hurdles, sustained lifestyle behaviour changes are possible. Lifestyle intervention trials can be difficult to design and carry out[36]. However, efforts should be made to overcome these barriers to carry out further studies or implement management strategies, given the enormous potential for improvement in health outcomes.

Despite very few multimodal lifestyle interventions for mental and physical health outcomes in PwMS, benefit has also been shown for people with other chronic diseases. In fact, management guidelines of other chronic diseases such as cardiovascular and cerebrovascular disease, and type-2 diabetes now routinely incorporate modification of lifestyle-related risk factors such as smoking cessation, diet, weight and physical activity[37–39], although uptake is variable[40]. Together with the results from our intervention, multimodal lifestyle intervention should be considered a potentially useful tool in the management of MS and other chronic diseases.

### Limitations

We have used the TIDieR guidelines to describe the intervention [16], however replicating a complex multimodal face-to-face intervention such as this would be challenging. An ongoing qualitative sub-study of participants in this study indicates that some participants started making lifestyle changes after signing up for the intervention but before completing the baseline survey of this study. While this may bias the results of our study it is likely that our results underestimate rather than overestimate the changes in health behaviours as participants may have increased healthy behaviours before baseline measures. All measures were self-reported, introducing potential measurement biases including recall bias. While QOL outcomes encompass energy, mood and affect, we did not specifically measure important health outcomes such as depression, anxiety, fatigue and cognition in an effort to minimise the burden of data collection on participants. However, further studies would benefit from including measures of these health outcomes as they are likely to be impacted by lifestyle change. As participants self-referred to the intervention, we did not have access to data from the participants’ physicians. Further, we did not collect data on health behaviours or outcomes from participants’ relatives, which would have strengthened our findings. There was a lack of control group for comparison, and regression to the mean may have influenced our results; participants may have self-selected for the intervention due to a recent relapse or worsening of symptoms. Furthermore, we don’t know the health status of participants who were lost to follow-up or did not take part in the study. Participants in our sample do not reflect the general population with MS; they were highly educated, had an interest in healthy lifestyle, and most were recently diagnosed. They were presumably highly motivated to make changes in lifestyle risk factors, as they spent considerable time and resources in order to participate in the intervention. While unlikely to be a representative sample, the reported impact of the intervention in this sample may be similar in the general MS population. The small sample size does not allow for sub-group analysis, and resulted in large confidence intervals. Blood lipid levels, vitamin D serum levels, and overall nutritional adequacy of the dietary and supplementation recommendations were not assessed. Our intervention was not informed by behaviour change theory and didn’t make use of specified behaviour change techniques. Further studies will benefit from using larger samples to increase precision of the estimates, collecting biological samples to assess changes in blood lipid profiles and nutritional deficiencies, and using behaviour change theory and techniques to maximise adherence to lifestyle changes.

## Conclusions

This study contributes to the literature regarding long-term follow-up after behavioural interventions in PwMS. The results provide evidence that lifestyle risk modification is feasible and sustainable over time, in a small self-selected and motivated sample of PwMS. These findings need to be replicated in larger, controlled studies.

## Supporting information

S1 Fig Questionnaire. (DOCX)

S2 Fig Intervention program. (DOCX)

S1 Table Baseline characteristics of the missing and non-missing data at each follow-up that are largely deterministic over time. (DOCX)

S2 Table Change in health outcomes using multiply imputed data. (DOCX)
