# Supplementary material for: Health outcomes and adherence to a healthy lifestyle after a multimodal intervention in people with multiple sclerosis: Three year follow-up
Source: PLoS One. 2018 May 23;13(5):e0197759. doi: 10.1371/journal.pone.0197759 (PMC5965868; doi:10.1371/journal.pone.0197759)
Supplement: S1 Fig — (DOCX) [file pone.0197759.s001.docx]

**Supplementary file 4. Questionnaire**

Participant Demographics

*** What is your gender?** Male/ Female

*** What is your year of birth?**

*** What is your marital status?**

| 1 | 2 | 3 | 4 | 5 |
| --- | --- | --- | --- | --- |
| Married | Single | Co-habiting/ partnered | Separated/Divorced | Widow(er) |

*** How many children (biological or other) do you have?**

*** What is your employment status?**

| 1 | 2 | 3 | 4 | 5 | 6 |
| --- | --- | --- | --- | --- | --- |
| Work  full-time | Work  part-time | Stay-at-home parent/ carer | Unemployed | Retired due to age | Retired due to MS |

*** What is the highest level of education you have completed?**

| 1 | 2 | 3 | 4 | 5 |
| --- | --- | --- | --- | --- |
| No formal schooling | Primary school | Secondary school | Vocational training (trade) | University (college) degree |

Multiple Sclerosis Diagnosis

Thank you, the survey is now complete.

NO/

UNSURE

*** Has a specialist diagnosed you with Multiple Sclerosis (MS)?** YES /

*** In which year did a specialist diagnose you with MS?**

(If unsure, please provide an approximate year)

*** In which year do you believe you first experienced symptoms of MS?**

(If unsure, please provide an approximate year)

*** Which type of MS were you first diagnosed with?** (Please circle the most appropriate response)

| 1 | 2 | 3 | 4 | 5 | 6 | 7 |
| --- | --- | --- | --- | --- | --- | --- |
| Benign | Relapsing-remitting | Primary progressive | Secondary progressive | Progressive relapsing | Unsure/Other | Not applicable |

*** Which type of MS do you have now?**

| 1 | 2 | 3 | 4 | 5 | 6 | 7 |
| --- | --- | --- | --- | --- | --- | --- |
| Benign | Relapsing-remitting | Primary progressive | Secondary progressive | Progressive relapsing | Unsure/Other | Not applicable |

*** How many relapses (diagnosed by your doctor) have you had within the last 12 months? (Leave blank if not applicable)**

*** How many relapses (diagnosed by your doctor) have you had within the last 5 years? (Leave blank if not applicable)**

Multiple Sclerosis Quality of Life Questionnaire (MSQOL-54)

**PLEASE ANSWER EVERY QUESTION BY CIRCLING THE MOST APPROPRIATE NUMBER**

**In general, would you say your health is:**

| Excellent | Very good | Good | Fair | Poor |
| --- | --- | --- | --- | --- |
| 1 | 2 | 3 | 4 | 5 |

**Compared to one year ago, how would you rate your health in general now?**

Much better than one year ago…………………….1

Somewhat better now than one year ago……..2

About the same…………………………………………….3

Somewhat worse now than one year ago……..4

Much worse now than one year ago……………..5

**The following questions are about activities you might do during a typical day.**

**Does your health limit you in these activities? If so, how much?**

|  | YES,  LIMITED A LOT | YES,  LIMITED A LITTLE | NO,  NOT LIMITED AT ALL |
| --- | --- | --- | --- |
| a) Vigorous activities, such as running, lifting heavy objects or participating in strenuous sports | 1 | 2 | 3 |
| b) Moderate activities, such as moving a table, pushing a vacuum cleaner, bowling or playing golf | 1 | 2 | 3 |
| c) Lifting or carrying groceries | 1 | 2 | 3 |
| d) Climbing several flights of stairs | 1 | 2 | 3 |
| e) Climbing one flight of stairs | 1 | 2 | 3 |
| f) Bending, kneeling, or stooping | 1 | 2 | 3 |
| g) Walking more than a mile | 1 | 2 | 3 |
| h) Walking several blocks | 1 | 2 | 3 |
| i) Walking one block | 1 | 2 | 3 |
| j) Bathing and dressing yourself | 1 | 2 | 3 |

**During the past 4 weeks, have you had any of the following problems with your work or other regular daily activities as a result of your physical health?**

|  | **YES** | **NO** |
| --- | --- | --- |
| a) Cut down on the amount of time you could spend on work or other activities | 1 | 2 |
| b) Accomplished less than you would like | 1 | 2 |
| c) Were limited in the kind of work or other activities | 1 | 2 |
| d) Had difficulty performing the work or other activities  (for example, it took extra effort) | 1 | 2 |

**During the past 4 weeks, have you had any of the following problems with your work or other regular daily activities as a result of any emotional problems (such as feeling anxious or depressed)?**

|  | **YES** | | **NO** |
| --- | --- | --- | --- |
| a) Cut down on the amount of time you could spend on work or other activities | 1 | | 2 |
| b) Accomplished less than you would like | 1 | | 2 |
| c) Didn’t do work or other activities as carefully as usual | 1 | 2 | |

**During the past 4 weeks, to what extent has your physical health or emotional problems interfered with your normal social activities with family, friends, neighbours or groups?**

| Not at all | Slightly | Moderately | Quite a bit | Extremely |
| --- | --- | --- | --- | --- |
| 1 | 2 | 3 | 4 | 5 |

**How much bodily pain have you had during the past 4 weeks?**

| None | Very mild | Mild | Moderate | Severe | Very severe |
| --- | --- | --- | --- | --- | --- |
| 1 | 2 | 3 | 4 | 5 | 6 |

**During the past 4 weeks, how much did pain interfere with your normal work (including both work outside the home and housework)?**

| Not at all | A little bit | Moderately | Quite a bit | Extremely |
| --- | --- | --- | --- | --- |
| 1 | 2 | 3 | 4 | 5 |

**These questions are about how you feel and how things have been with you during the past 4 weeks. For each question, please give the one answer that comes closest to the way you have been feeling.**

**How much of the time during the past 4 weeks…**

|  | All of  the time | Most of  the time | A good bit of the time | Some of  the time | A little of the time | None of the time |
| --- | --- | --- | --- | --- | --- | --- |
| a) Did you feel full of pep (energy/bounce)? | 1 | 2 | 3 | 4 | 5 | 6 |
| b) Have you been a very nervous person? | 1 | 2 | 3 | 4 | 5 | 6 |
| c) Have you felt so down in the dumps that nothing could cheer you up? | 1 | 2 | 3 | 4 | 5 | 6 |
| d) Have you felt calm and peaceful? | 1 | 2 | 3 | 4 | 5 | 6 |
| e) Did you have a lot of energy? | 1 | 2 | 3 | 4 | 5 | 6 |
| f) Have you felt downhearted and blue? | 1 | 2 | 3 | 4 | 5 | 6 |
| g) Did you feel worn out? | 1 | 2 | 3 | 4 | 5 | 6 |
| h) Have you been a happy person? | 1 | 2 | 3 | 4 | 5 | 6 |
| i) Did you feel tired? | 1 | 2 | 3 | 4 | 5 | 6 |
| j) Did you feel rested on waking in the morning? | 1 | 2 | 3 | 4 | 5 | 6 |

**During the past 4 weeks, how much of the time has your physical health or emotional problems interfered with your social activities (like visiting friends, relatives etc)?**

| All of the time | Most of the time | Some of the time | A little of the time | None of the time |
| --- | --- | --- | --- | --- |
| 1 | 2 | 3 | 4 | 5 |

**How TRUE or FALSE is each of the following statements for you?**

|  | Definitely true | Mostly true | Not sure | Mostly false | Definitely false |
| --- | --- | --- | --- | --- | --- |
| a) I seem to get sick a little easier than other people | 1 | 2 | 3 | 4 | 5 |
| b) I am as healthy as anybody I know | 1 | 2 | 3 | 4 | 5 |
| c) I expect my health to get worse | 1 | 2 | 3 | 4 | 5 |
| d) My health is excellent | 1 | 2 | 3 | 4 | 5 |

**How much of the time during the past 4 weeks….**

|  | All of  the time | Most of  the time | A good bit of the time | Some of  the time | A little of the time | None of the time |
| --- | --- | --- | --- | --- | --- | --- |
| a) Were you discouraged by your health problems? | 1 | 2 | 3 | 4 | 5 | 6 |
| b) Were you frustrated by your health? | 1 | 2 | 3 | 4 | 5 | 6 |
| c) Was your health a worry in your life? | 1 | 2 | 3 | 4 | 5 | 6 |
| d) Did you feel weighed down by your health problems? | 1 | 2 | 3 | 4 | 5 | 6 |

| **a)** Have you had difficulty concentrating and thinking? | 1 | 2 | 3 | 4 | 5 | 6 |
| --- | --- | --- | --- | --- | --- | --- |
| b) Did you have trouble keeping your attention on an activity for long? | 1 | 2 | 3 | 4 | 5 | 6 |
| c) Have you had trouble with your memory? | 1 | 2 | 3 | 4 | 5 | 6 |
| d) Have others, such as family members or friends, noticed that you have trouble with your memory or problems with your concentration? | 1 | 2 | 3 | 4 | 5 | 6 |

**The next set of questions are about your sexual function and your satisfaction with your sexual function. Please answer as accurately as possible about your function during the past 4 weeks only. If you feel that these questions are not applicable to you or you feel uncomfortable answering these, please feel free to leave these questions blank.**

**How much of a problem was each of the following for you during the past 4 weeks?**

| **MEN** | Not a problem | A little of a problem | Somewhat of a problem | Very much a problem |
| --- | --- | --- | --- | --- |
| a) Lack of sexual interest | 1 | 2 | 3 | 4 |
| b) Difficulty getting or keeping an erection | 1 | 2 | 3 | 4 |
| c) Difficulty having orgasm | 1 | 2 | 3 | 4 |
| d) Ability to satisfy sexual partner | 1 | 2 | 3 | 4 |

| **WOMEN** | Not a problem | A little of a problem | Somewhat of a problem | Very much a problem |
| --- | --- | --- | --- | --- |
| a) Lack of sexual interest | 1 | 2 | 3 | 4 |
| b) Inadequate lubrication | 1 | 2 | 3 | 4 |
| c) Difficulty having orgasm | 1 | 2 | 3 | 4 |
| d) Ability to satisfy sexual partner | 1 | 2 | 3 | 4 |

**Overall, how satisfied were you with your sexual function during the past 4 weeks?**

Very satisfied…………………………………………………….1

Somewhat satisfied…………………………………………..2

Neither satisfied nor dissatisfied……………………….3

Somewhat dissatisfied………………………………………4

Very dissatisfied………………………………………………..5

**During the past 4 weeks, to what extent have problems with your bowel or bladder function interfered with your normal social activities with family, friends, neighbours or groups?**

| Not at all | Slightly | Moderately | Quite a bit | Extremely |
| --- | --- | --- | --- | --- |
| 1 | 2 | 3 | 4 | 5 |

**During the past 4 weeks, how much did pain interfere with your enjoyment of life?**

| Not at all | Slightly | Moderately | Quite a bit | Extremely |
| --- | --- | --- | --- | --- |
| 1 | 2 | 3 | 4 | 5 |

**Overall, how would you rate your own quality-of-life?**

(Circle one number on the scale below)

|  |  |  |  |  |  |  |  |  |  |
| --- | --- | --- | --- | --- | --- | --- | --- | --- | --- |

0 1 2 3 4 5 6 7 8 9 10

Best possible quality of life

Worst possible quality of life:

*as bad as or worse than being dead*

**Which best describes how you feel about your life as a whole?**

Terrible……………………………………………..1

Unhappy…………………………………………….2

Mostly dissatisfied……………………………..3

Mixed – about equally

satisfied and dissatisfied…………………….4

Mostly satisfied………………………………….5

Pleased………………………………………………6

Delighted……………………………………………7

Diet Habits Questionnaire

Please complete the following questions by selecting the best option that applies to you.

**How many days a week do you eat a high fibre breakfast cereal? (e.g. rolled oats, Weet-bix, Allbran, untoasted muesli)**

- Six or more times per week
- Three to five times per week
- Once or twice per week
- Less than once per week
- Never, or hardly ever

**How often do you eat or use wholemeal or wholegrain bread in preference to white bread?**

- Always
- Usually
- Occasionally
- Rarely
- Never
- N/A (I don’t eat bread)

**How often do you eat cereal e.g. pasta, rice, noodles, cous cous, as part of your main meal?**

- Five or more times per week
- Three to four times per week
- Once or twice per week
- Rarely
- Never

**How many serves of vegetables do you eat in a typical day? (One serve is equal to half a cup of cooked vegetables or a cup of salad).**

- Five or more serves
- Three to four serves
- One or two serves
- Less than one serve a day
- None

**How many different types of vegetables would you eat on a typical day?**

- Five or more types
- Four types
- Three types
- One or two types
- None

**How many times a week do you eat two or more pieces of fruit a day?**

- Six or more times per week
- Three to five times per week
- Once or twice per week
- Less than once per week
- Never, or hardly ever

**How many days a week do you eat legumes? (eg. chick peas, lentils, split peas, kidney beans etc)**

- Two or more times per week
- Once per week
- Once per fortnight
- Once per month
- Never

**How often do you eat raw nuts or seeds such as pepitas, sunflower seeds and linseeds?**

- Five or more times per week
- Three to four times per week
- Once or twice per week
- Rarely
- Never

**When having milk, yoghurt or cheese (from dairy sources), how often do you eat or use reduced-fat or low fat products in preference to regular products?**

- Always
- Usually
- Occasionally
- Rarely
- Never
- I don’t consume dairy products

**How many days a week do you eat fish?**

- Three or more days per week
- Two days per week
- Once per week
- Less than once per week
- Never, or hardly ever

**If you use a spread on bread or cracker biscuits, which type of spread would you usually use?**

- Margarine (poly/monounsaturated/sterol)
- Avocado
- Cream cheese
- Butter
- I don’t use spreads

**How often do you eat processed meats (eg. bacon, sausages, salami, ham, frankfurts, pate)?**

- Four or more times per week
- Two or three times per week
- Once per week
- Less than once per week
- Never, or hardly ever
- I don’t eat processed meats

**What type of salad dressing do you normally use?**

- Mono/poly unsaturated** oil base
- Reduced fat commercial dressing
- Full fat or creamy commercial dressing
- I don’t use dressing on salads

**What type of cooked sauces do you normally use? (You may select more than one).**

- Tomato/Vegetable-based sauces
- Reduced-fat, milk based
- Gravy from commercial powder
- Gravy from pan dripping
- Cream or full cream milk based
- Sauces with coconut milk
- I don’t use cooked sauces
- Other (please specify)

**How often do you trim all the visible fat off the meat you eat (or purchase pre-trimmed meat) and remove the skin from chicken before cooking?**

- Always
- Usually
- Occasionally
- Rarely
- Never
- I don’t eat meat

**When cooking, which of the following cooking fats do you normally use?**

- Butter
- Solid frying fat
- Vegetable oil*
- Mono/poly unsaturated oils **
- Sterol margarine
- Spray oil
- I don’t use fat in cooking

* Vegetable oil – eg. coconut, palm

** Mono-unsaturated – eg. olive, canola, pecan, almond, peanut

** Polyunsaturated – eg. corn, soy, cottonseed, safflower, sunflower, walnut, flaxseed, fish.

**Which of the following cooking methods do you commonly use when cooking? (You may select more than one).**

- Steaming
- Poaching
- Microwaving
- Casseroles
- Grilling
- Stir fries
- Dry roasting
- Deep frying
- Shallow frying
- Roasting in fat

**How often do you eat pastries, cake, sweet biscuits or croissants?**

- Six or more times per week
- Three to five times per week
- Once or twice per week
- Less than once per week
- Never, or hardly ever

**How many days a week do you eat take-away style foods such as fried or BBQ chicken, fish and chips, Chinese, pizza, hamburgers etc?**

- Five or more times per week
- Three to four times per week
- Once or twice a week
- Less than once per week
- Never, or hardly ever

**Which of the following foods do you eat most often as snacks between meals?**

- Chocolate bars
- Crisps/chips
- Sweet biscuits, cake
- Low fat yoghurts (dairy)
- Olives, raw nuts, seeds
- Fruit, dried fruit
- Fruit bread, English muffins
- I don’t snack between meals
- Other (please specify)

**Do you currently smoke tobacco products?**

- Yes
- No, I have never smoked
- No, but I used to smoke

**If you used to smoke tobacco products, how long ago did you quit?**

**If you currently smoke, on average, how many cigarettes do you smoke each day?**

### International Physical Activity Questionnaire (IPAQ)

**Please answer each question below even if you do not consider yourself to be an active person or your MS limits your ability to exercise. Think about the activities you do at work, as part of your house and yard work, to get from place to place, and in your spare time for recreation, exercise or sport.**

During the **last 7 days**, on how many days did you do **vigorous** physical activities for more than 10 minutes at a time (eg. heavy lifting, digging, aerobics, or fast bicycling)?

_____ **days per week**

How much time on average did you usually spend on one of those days doing vigorous physical activities?

_____ **hours per day _____ minutes per day**

OR

None

During the **last 7 days**, on how many days did you do **moderate** physical activities for more than 10 minutes at a time (eg. carrying light loads, bicycling at a regular pace, or doubles tennis)? Do not include walking.

How much time on average did you usually spend on one of those days doing moderate physical activities?

_____ **hours per day _____ minutes per day**

_____ **days per week**

OR

None

During the **last 7 days**, on how many days did you **walk** for at least 10 minutes at a time? This could include walking at work and at home, traveling from place to place and any other walking that you did for exercise or leisure.

How much time on average did you usually spend walking on one of those days?

_____ **hours per day _____ minutes per day**

_____ **days per week**

OR

None

During the **last 7 days**, how much time in total did you usually spend **sitting** on a **week day**?

This could include time spent in a car, or on public transport, sitting at a desk or computer, visiting friends, meal times, reading, or sitting or lying down to watch television (do not include sleeping).

_____ **hours per day** _____ **minutes per day**

**On average in the past 12 months, how often have you meditated?**

- Never
- Less than once per week
- 1-2 times per week
- 3-4 times per week
- 5-6 times per week
- Every day

**On average, for how long do you meditate (in minutes) each time?**

**Do you take a vitamin D supplement?**

YES / NO (If ‘no’ skip next 2 questions)

**What dose (in international units IU) of vitamin D do you take?**

**How often do you take this dose of vitamin D?**

- Daily
- Every second day
- Weekly
- Monthly
- Other (please specify)

**Which type(s) of Omega-3 supplements do you take?**

- I don’t take Omega-3 supplements
- Fish oil
- Flaxseed oil
- High potency fish oil
- Other (please specify)

**In the past 12 months, what total dose of Omega-3 supplements (as standard strength fish oil or flaxseed oil measured in grams, mls or number of capsules) do you take on average per day?**

- 0
- 1-5
- 6-10
- 11-15
- 16-20
- 21-25
- 26-30
- More than 30

**In the past 12 months, how much additional Omega-3 (measured as grams of fish oil or flaxseed oil) do you feel you get on average per day in food (eg. fish, seafood, flaxseeds, walnuts, pecans)**

- 0
- 1-5
- 6-10
- 11-15
- 16-20
- 21-25
- 26-30
- More than 30
- Unsure

**At present, do you use any medications for the management of MS?** YES / NO

**If so, please indicate which medication(s) you presently use to manage MS:**

- Steroids (Prednisolone, Prednisone)
- Interferons (Avonex, Rebif, Betaferon)
- Glatiramer (Capaxone)
- Natalizumab (Tysabri)
- Mitoxantrone (Novantrone)
- Immunoglobulins (Gamma globulin)
- Methotrexate (Folex, Rheumatrex)
- Azathioprine (Imuran)
- Cyclophosphamide (Cytoxan, Revimmune)
- Fingolimod (Gilenya)
- Cladribine (Movectro, Movectra)
- Lose-dose Naltrexone (LDN)
- Minocycline (Minomycin)
- Baclofen (Kemnstro, Lioresal)
- Fampridine (Amprya)
- Other (please specify)

**Please list any other medications you take to manage symptoms of MS such as fatigue, depression or pain, including non-prescription medications or herbal remedies.**

Multiple Sclerosis Impact Scale (MSIS-29)

**The following questions ask for your views about the impact of MS on your day-to-day life during the past two weeks. For each statement, please select the response that best describes your situation.**

**In the past two weeks, how much has your MS limited your ability to…**

|  | Not at all | A little | Moderately | Quite a bit | Extremely |
| --- | --- | --- | --- | --- | --- |
| a) Do physically demanding tasks? | 1 | 2 | 3 | 4 | 5 |
| b) Grip thing tightly? (eg. Turn on taps) | 1 | 2 | 3 | 4 | 5 |
| c) Carry things? | 1 | 2 | 3 | 4 | 5 |

**In the past two weeks, how much have you been bothered by…**

|  | Not at all | A little | Moderately | Quite a bit | Extremely |
| --- | --- | --- | --- | --- | --- |
| a) Problems with your balance? | 1 | 2 | 3 | 4 | 5 |
| b) Difficulties moving about indoors? | 1 | 2 | 3 | 4 | 5 |
| c) Being clumsy? | 1 | 2 | 3 | 4 | 5 |
| d) Stiffness? | 1 | 2 | 3 | 4 | 5 |
| e) Heavy arms and/or legs? | 1 | 2 | 3 | 4 | 5 |
| f) Tremor of your arms and/or legs? | 1 | 2 | 3 | 4 | 5 |
| g) Spasms in your limbs? | 1 | 2 | 3 | 4 | 5 |
| h) Your body not doing what you want it to do? | 1 | 2 | 3 | 4 | 5 |
| i) Having to depend on others to do things for you? | 1 | 2 | 3 | 4 | 5 |
| j) Limitations in your social and leisure activities at home? | 1 | 2 | 3 | 4 | 5 |

**In the past two weeks, how much have you been bothered by…**

|  | Not at all | A little | Moderately | Quite a bit | Extremely |
| --- | --- | --- | --- | --- | --- |
| k) Being stuck at home more than you would like to be? | 1 | 2 | 3 | 4 | 5 |
| l) Difficulties using your hands in everyday tasks? | 1 | 2 | 3 | 4 | 5 |
| m) Having to cut down on the amount of time you spent on work or other daily activities? | 1 | 2 | 3 | 4 | 5 |
| n) Problems using transport (eg. Car, bus, train, taxi, etc.)? | 1 | 2 | 3 | 4 | 5 |
| o) Taking longer to do things? | 1 | 2 | 3 | 4 | 5 |
| p) Difficulty doing things spontaneously (eg. Going out on the spur of the moment)? | 1 | 2 | 3 | 4 | 5 |
| q) Needing to go to the toilet urgently? | 1 | 2 | 3 | 4 | 5 |

**Please take the time to check all questions have been answered or a comment written.**

**Thank you for taking the time to complete the Overcoming Multiple Sclerosis questionnaire. Your involvement in this worthwhile study is greatly appreciated. If you have any questions about the questionnaire or project please contact our research officer on (03) 98344 2173.**
