# Supplementary material for: Health outcomes and adherence to a healthy lifestyle after a multimodal intervention in people with multiple sclerosis: Three year follow-up
Source: PLoS One. 2018 May 23;13(5):e0197759. doi: 10.1371/journal.pone.0197759 (PMC5965868; doi:10.1371/journal.pone.0197759)
Supplement: S2 Fig — (DOCX) [file pone.0197759.s002.docx]

**Supplementary File 2. Intervention program**

| ***Daily Program*** |  |  |
| --- | --- | --- |
| 7.15 am | Wake up bell |  |
| 8.30 am | Breakfast |  |
| 11.15-11.45am | Morning Tea |  |
| 1.00 pm  4.00-4.30pm | Lunch & Free Time  Afternoon Tea |  |
| 6.30 pm | Dinner |  |
|  |  |  |
| ***Monday*** |  |  |
| 9.30 - 10.30am | Guests arrive |  |
| 11.00am - 1.00pm | Welcome & Introductions, Meditation | Facilitators 1, 2 and 3 |
| 2.30 - 4.00pm | Basic Anatomy, Causes/Theories of MS | Facilitators 1&2 |
| 4.30 - 5.50pm | Self help Interventions | Facilitators 1&2 |
| 6.00 - 6.30pm | Meditation | Facilitator 1 |
| 8.00pm | Free Night |  |
| ***Tuesday*** | | |
| 9.15 - 10.30am | Meditation I | Facilitator 4 |
| 10.30 - 11.15am | Qigong | Facilitator 4 |
| 11.45 - 1.00pm | Meditation 2 | Facilitator 4 |
| 3.00 – 4.30pm | Medications in MS | Facilitators 1&2 |
| 5.00 – 6.00pm | Reflections | Facilitators 1&2 |
| 6.00 – 6.30pm | Meditation | Facilitator 1 |
| 8.00pm | Mindfulness and Music | Musician |
| ***Wednesday*** | | |
| 9.15 - 10.30am | Food 1 – the best foods & what to avoid | Facilitators 1&2 |
| 10.30 - 11.15am | Qigong | Facilitator 4 |
| 11.35am | Photo | Programs |
| 11.45am - 1.00pm | Supplements & Sunlight | Facilitators 1&2 |
| 3.00 – 4.00pm | Food 2 | Facilitators 1&2 |
| 4.30 – 5.45pm | a) Affirmations and Imagery for people with MS | Facilitator 4 |
|  | b) Small group discussion for partners | Facilitator 3 |
| 6.00 - 6.30pm | Meditation | Facilitator 3 |
| 8.00pm | *Film Night* |  |
| ***Thursday*** | | |
| 9.15 - 10.30am | Healthy Emotions I | Facilitators 4&5 |
| 10.30 - 11.15am | Qigong | Facilitator 4 |
| 11.45am - 1.00pm | Healthy Emotions II | Facilitators 4&5 |
| 3.00 – 4.00pm | Forgiveness | Facilitators 4&5 |
| 5.00 – 6.30pm | MS & Spirituality | Facilitator 3 |
| 8.00pm | Laughter – The Best Medicine | Facilitator 3 |
| ***Friday*** | | |
| 9.30 - 10.45am | Recommendation, planning for the future | Facilitator 1&3 |
| 10.45 - 11.15am | Morning Tea |  |
| 11.15am - 1.00pm | Review of program, assessments, farewell | Facilitator 1&3 |
| 1.00 - 2.00pm | Lunch & Departure |  |
