# Supplementary material for: Health outcomes and adherence to a healthy lifestyle after a multimodal intervention in people with multiple sclerosis: Three year follow-up
Source: PLoS One. 2018 May 23;13(5):e0197759. doi: 10.1371/journal.pone.0197759 (PMC5965868; doi:10.1371/journal.pone.0197759)
Supplement: S1 Table — (DOCX) [file pone.0197759.s003.docx]

**Supplementary Table 1: Baseline characteristics of the missing and non-missing data at each follow-up that are largely deterministic over time**

| **Variable** | **1 year follow-up** | | | **3 year follow-up** | | |  |  |
| --- | --- | --- | --- | --- | --- | --- | --- | --- |
|  | Non-missing | | Missing | Non-missing | | Missing | |  |
| Age (mean, sd) | 43.9 (10.2) | 44.63 (11.6) | | | 44.14 (10.4) | 43.47 (11.0) | | |
| Gender, Male (N, %) | 22/76 (29.0.) | 4/19 (21.1) | | | 25/78 (32.1) | 1/17 (5.9) | | |
| University level education (N,%) | 18/76 (23.7) | 9/19 (47.4) | | | 21/78 (26.9) | 6/17 (35.3) | | |
