# Supplementary material for: Health outcomes and adherence to a healthy lifestyle after a multimodal intervention in people with multiple sclerosis: Three year follow-up
Source: PLoS One. 2018 May 23;13(5):e0197759. doi: 10.1371/journal.pone.0197759 (PMC5965868; doi:10.1371/journal.pone.0197759)
Supplement: S2 Table — (DOCX) [file pone.0197759.s004.docx]

**Supplementary Table 2: Change in health outcomes using multiply imputed data**

| **Health outcomes** | | **Mean difference** | **95% CI** | **p-value** |
| --- | --- | --- | --- | --- |
| Level of disability (MSIS) | Baseline | Reference |  |  |
|  | I year | 0.9 | (0.9,1.0) | 0.01 |
|  | 3 year | 0.9 | (0.9,1.0) | 0.03 |
| Mental health QOL | Baseline | Reference |  |  |
|  | I year | 8.6 | (4.4,12.8) | <0.001 |
|  | 3 year | 8.4 | (4.2,12.5) | <0.001 |
| Physical health QOL | Baseline | Reference |  |  |
|  | I year | 7.9 | (4.8,11.0) | <0.001 |
|  | 3 year | 8.3 | (4.9,11.8) | <0.001 |
| Any relapses in previous calendar year | Baseline | Reference |  |  |
|  | I year |  |  |  |
|  | 3 year |  |  |  |
